# Hydrogeochemical controls on brook trout spawning habitats in a coastal stream

**DOI:** 10.5194/hess-22-6383-2018

**Published:** 2018

**Authors:** Martin A. Briggs, Judson W. Harvey, Stephen T. Hurley, Donald O. Rosenberry, Timothy McCobb, Dale Werkema, John W. Lane

**Affiliations:** 1 U.S. Geological Survey, Hydrogeophysics Branch, 11 Sherman Place, Unit 5015, Storrs, CT 06269, USA; 2 U.S. Geological Survey, Water Cycle Branch, M.S. 430, Reston, VA 20192, USA; 3 Massachusetts Division of Fisheries and Wildlife, 195 Bournedale Road, Buzzards Bay, MA 02532, USA; 4 U.S. Geological Survey, National Research Program, M.S. 406, Bldg. 25, DFC, Lakewood, CO 80225, USA; 5 U.S. Geological Survey, 10 Bearfoot Road, Northborough, MA 01532, USA; 6 U.S. Environmental Protection Agency, Office of Research and Development, National Exposure Research Laboratory, Exposure Methods & Measurement Division, Environmental Chemistry Branch, Las Vegas, NV 89119 USA

## Abstract

Brook trout *(Salvelinus fontinalis)* spawn in fall and overwintering egg development can benefit from stable, relatively warm temperatures in groundwater-seepage zones. However, eggs are also sensitive to dissolved oxygen concentration, which may be reduced in discharging groundwater (i.e., seepage). We investigated a 2 km reach of the coastal Quashnet River in Cape Cod, Massachusetts, USA, to relate preferred fish spawning habitats to geology, geomorphology, and discharging groundwater geochemistry. Thermal reconnaissance methods were used to locate zones of rapid groundwater discharge, which were predominantly found along the central channel of a wider stream valley section. Pore-water chemistry and temporal vertical groundwater flux were measured at a subset of these zones during field campaigns over several seasons. Seepage zones in open-valley sub-reaches generally showed suboxic conditions and higher dissolved solutes compared to the underlying glacial outwash aquifer. These discharge zones were cross-referenced with preferred brook trout redds and evaluated during 10 years of observation, all of which were associated with discrete alcove features in steep cutbanks, where stream meander bends intersect the glacial valley walls. Seepage in these repeat spawning zones was generally stronger and more variable than in open-valley sites, with higher dissolved oxygen and reduced solute concentrations. The combined evidence indicates that regional groundwater discharge along the broader valley bottom is predominantly suboxic due to the influence of near-stream organic deposits; trout show no obvious preference for these zones when spawning. However, the meander bends that cut into sandy deposits near the valley walls generate strong oxic seepage zones that are utilized routinely for redd construction and the overwintering of trout eggs. Stable water isotopic data support the conclusion that repeat spawning zones are located directly on preferential discharges of more localized groundwater. In similar coastal systems with extensive valley peat deposits, the specific use of groundwater-discharge points by brook trout may be limited to morphologies such as cutbanks, where groundwater flow paths do not encounter substantial buried organic material and remain oxygen-rich.

## 1 Introduction

The heat tracing of water can be used to map a distribution of spatially focused, or “preferential”, groundwater-discharge zones throughout surface water systems at times of contrast between the surface and groundwater temperature. The measurement of the water temperature from the reach to watershed scale is now possible using thermal infrared and fiber-optic distributed temperature sensing (FO-DTS) methodology (Dugdale, 2016; Hare et al., 2015; Steel et al., 2017). Remote infrared data collection throughout the river corridor has been enabled by handheld cameras, piloted aircraft, and the rapidly evolving capabilities of unmanned aerial systems. Researchers are capitalizing on the ongoing refinement of these technologies to identify zones of focused groundwater seepage to streams in order to map potential discrete preferential cold-water fish habitats such as summer thermal refugia (Dugdale et al., 2015). However, surface thermal surveys alone do not indicate groundwater flow path dynamics or the suitability of an interface aquatic habitat (Briggs et al., 2018a).

For example, dissolved oxygen (DO) concentration must be sufficiently high for cold groundwater seepage to provide support for fish life processes at the direct point of discharge to surface water (Ebersole et al., 2003), which is not apparent from thermal analysis alone. During warm summer periods in systems with suboxic groundwater, cold-water fish species such as salmonids can face a tradeoff between occupying discrete zones of preferred water temperatures with near-lethal DO levels and stream sections that are too warm for long-term survival (Matthews and Berg, 1997). The use of groundwater upwelling zones as thermal refugia is further complicated by competition with aggressive invasive species (to the northeastern USA) such as brown trout, which compete with native trout for resources (Hitt et al., 2017). Streams at higher elevations may support the persistence of reach-scale cold-water habitats where point-scale thermal refugia are not needed under current climatic conditions, serving as vital “climate refugia” against rising air temperatures (Isaak et al., 2015). In systems with reliably cold channel water in summer, which can also exist at low elevations when heavily influenced by discharging groundwater, salmonid fish may directly use groundwater-seepage zones for spawning rather than thermal refuge.

Brook trout *(Salvelinus fontinalis)* are a species of char that are native to eastern North America, from Georgia to Québec (MacCrimmon and Campbell, 1969). Populations have been stressed by warming temperatures and reduced water quality, particularly in low-elevation areas (Hudy et al., 2008). Stream network-scale tracking of fish has indicated that the brook trout directly utilize stream confluence mixing zones and preferential groundwater discharge to survive warm summer periods (Baird and Krueger, 2003; Petty et al., 2012; Snook et al., 2016). Additionally, brook trout spawn in the fall, and eggs deposited in redds develop over the winter before hatching in spring (Cunjak and Power, 1986). Oxygen use by the shallow buried embryos increases over the period of development (Crisp, 1981); therefore, DO concentration is a critical parameter of the pore water in which the eggs are bathed. Several studies have demonstrated the importance of hyporheic downwelling in increasing shallow oxygen concentrations, including for salmonid redds, where deeper stream-bed pore water is generally reduced in DO (e.g., Buffington and Tonina 2009; Cardenas et al., 2016; Harvey et al., 2013). Fine sediments can reduce the efficacy of hyporheic DO exchange in spawn zones (Obruca and Hauer, 2016) and are actively cleared by trout during the spawning process (Montgomery et al., 1996).

The importance of hyporheic exchange to salmonid spawning may be limited in the lowland streams that are expected to harbor native cold-water species in the 21st century, namely those with strong groundwater influence. Groundwater upwelling reduces the penetration of the hyporheic flow from surface water (Cardenas and Wilson, 2006) and may shut down hyporheic flushing in redds (Cardenas et al., 2016). While hyporheic exchange introduces oxygenated channel water into the shallow stream bed, the downward advection of heat associated with nearfreezing surface water in winter will also cool streambed sediments (Geist et al., 2002), potentially impairing egg development. Coaster brook trout, a life-history variant of native brook trout exhibiting potadromous migrations within the Great Lakes, have been shown to specifically prefer groundwater-discharge zones for building redds (Van Grinsven et al., 2012). The development of trout in winter has been found to positively correlate with warmer stream water temperatures as influenced by groundwater seepage (French et al., 2017). Therefore, spatially discrete groundwater-discharge zones with adequate DO may form preferred brook trout spawning habitats (Curry et al., 1995).

Multiscale physical and biogeochemical factors influence temperature and DO concentrations along groundwater flow paths. In river valleys, discharge to the surface water of locally recharged groundwater is expected to emanate from more shallow, lateral flow paths controlled by the local topography (Modica, 1999; Winter et al., 1998). Shallow groundwater flow paths, particularly those within approximately 5 m of the land surface, will be more sensitive to annual air temperature patterns and long-term warming trends due to strong vertical conductive heat exchanges (Kurylyk et al., 2015b). The distance of seeps from upgradient groundwater recharge zones will also affect seepage temperature dynamics and associated aquatic ecosystems due to future changes in surface and recharge temperatures (Burns et al., 2017). Therefore, characterizing the hydrogeochemical attributes of discharging groundwater flow paths is critical in understanding the thermal stability of current and future point-scale preferential brook trout habitats (Briggs et al., 2018a). The complimentary methodology of geophysical remote sensing, geochemical sampling, and vertical bed temperature time series can indicate the physical and chemical properties of groundwater flow paths that source preferential discharge zones utilized routinely by fish for spawning.

Coarse-grained mineral-dominated aquifers with little fine particulate organic matter and low dissolved organic carbon supplies tend to result in generally oxic groundwater conditions (Back et al., 1993). The sandy surficial aquifer of Cape Cod, where our investigation took place, is a classic example of a mineral soil-dominated flow system (Frimpter and Gay, 1979). The flow of groundwater through near-stream organic deposits, however, can result in inverted redox gradients toward the upwelling interface, such that groundwater discharged to surface water is reduced in DO (Seitzinger et al., 2006). In sandy glacial terrain with superimposed peatland deposits, the specific flow patterns of groundwater to surface water in relation to buried peat will influence the groundwater-discharge biogeochemistry. Krause et al. (2013) found that stream-bed groundwater seepage was strongly reduced in DO in zones with peat deposits, likely due to an increase in both near-stream residence time and localized sources of dissolved organic carbon.

Interdisciplinary collaborations between physical and biological scientists are useful to better understand how cold-water species utilize the stream habitat influenced by groundwater discharge and the larger landscape-scale controls on discharge characteristics. While previous hydrogeological research in the coastal stream used for this study had focused on locating and quantifying discrete groundwater discharge (e.g., “cold anomalies”, Hare et al., 2015; Rosenberry et al., 2016), here we endeavor to understand the hydraulic and biogeochemical controls on seepage zone distribution utilized directly by native brook trout. In this groundwater-dominated stream (e.g., likely climate refugia), brook trout do not need to occupy discrete inflows for summer thermal refugia but do favor certain upwelling zones for fall spawning. We compare over a decade of visual survey and electronic fish passive integrated transponder (PIT)-tag dropout data regarding repeat brook trout spawning locations to a comprehensive physical and chemical characterization of groundwater-seepage zones across 2 km of stream in order to do the following:

1. identify repeat brook trout spawning locations and determine if they are directly associated with the preferential discharge of groundwater through interface sediments, and
2. develop a hydrogeochemical characterization of trout-preferred groundwater-discharge zones that can aid in their identification in other less-studied systems and potential inclusion in stream habitat restoration efforts.

## 2 Site description and previous hydrogeologic characterization

Cape Cod is a peninsula in southeastern coastal Massachusetts, USA, composed primarily of highly permeable unconsolidated glacial moraine and outwash deposits. The largest of the Cape Cod sole-source aquifers occupies a western (landward) section of the peninsula (LeBlanc et al., 1986) and is incised by several linear valleys that drain groundwater south to the Atlantic Ocean via baseflow-dominated streams. Strong groundwater discharge to one such stream, the Quashnet River, supports a relatively stable flow regime that has averaged 0.49 ± 0.15(SD)m^3^ s^−1^ from 1986 to 2015 (Rosenberry et al., 2016). The lower Quashnet River emerges from a narrow sand and gravel valley into a broader area with well-defined lateral floodplains. Historical cranberry farming practices, abandoned in the 1950s, have modified the stream corridor (Barlow and Hess, 1993). Primary modifications included the straightening of the main channel (reducing natural sinuosity), installation of flood-control structures, incision of shallow groundwater drainage ditches in the lateral peatland floodplain, and widespread application of sand to the floodplain surface. The current bank-full width of the main channel averages approximately 4 m.

The Quashnet River has long been recognized as a critical habitat for a naturally reproducing population of native sea-run brook trout (Mullan, 1958) with a genetically distinct population (Annett et al., 2012). Efforts to restore trout habitats by the group Trout Unlimited and others have been ongoing for over 40 years (Barlow and Hess, 1993). These efforts include the removal of flood-control structures, the planting of trees along the main channel, and the addition of wood structures to stabilize banks and provide cover from airborne predators. Furthermore, the Commonwealth of Massachusetts purchased 12.5 ha in 1956 and an additional 146 ha along the lower Quashnet River in 1987 and 1988 to protect the area from development. The Massachusetts Division of Fisheries and Wildlife has been monitoring trout populations since 1988 and their movement since 2007.

The groundwater influence on stream temperature is pronounced, particularly over the 2 km reach above the U.S. Geological Survey gage no. 011058837, below which the stream stage is tidally affected. Ambient regional groundwater temperature is approximately 11 °C (Briggs et al., 2014), and strong conductive and advective exchange with the proximal aquifer maintains the surface water temperature well below the lethal threshold for brook trout (maximum weekly average temperature > 23.3 °C, Wehrly et al., 2007). Therefore, point-scale thermal refugia are not a current concern in this system, as the stream supports a system-scale cold-water habitat that is likely to persist into the future and serve as warming “climate refugia” (Briggs et al., 2018a). In winter, seepage zones can be located as relatively warm anomalies, increasing and buffering surface water temperatures from ambient atmospheric influence.

**Figure 1.**
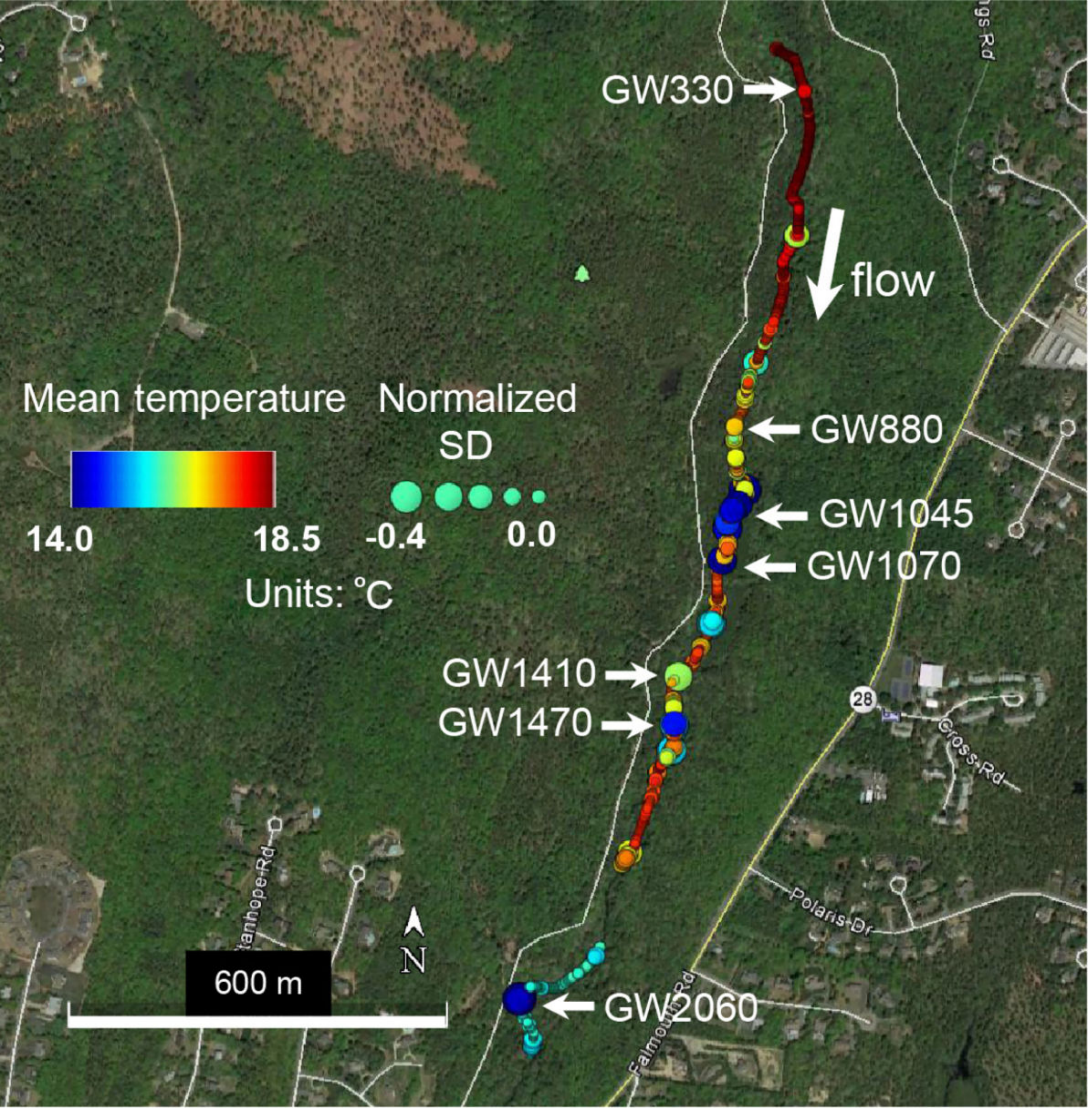
Fiber-optic distributed temperature data collected along the stream channel sediment-water interface over two days in July 2013 are summarized here using mean temperature (color) and temperature standard deviation normalized to known non-seepage locations (size). Locations of reduced mean temperature and the standard deviation of temperature can indicate zones of preferential groundwater upwelling. A subset of these apparent upwelling zones (labeled “GW” followed by the distance from upper reach boundary in meters) with varied thermal statistics was chosen for direct pore-water sampling and quantitative seepage measurements. This figure was modified from Rosenberry et al. (2016).

Previous work has measured relatively large net gains in streamflow over the lower Quashnet River (Barlow and Hess, 1993; Rosenberry et al., 2016), which are attributed to groundwater discharge through direct stream-bed seepage and the harvesting of groundwater from the floodplain platform via relic agricultural drainage ditches. Deployments of fiber-optic temperature sensing (FO-DTS) cables along the thalweg stream-bed interface indicate that the greatest density of focused seepage zones occurs along the broader valley area, approximately 1 km upstream of the U.S. Geological Survey gage (Fig. 1). This zone coincides with the largest gains in net streamflow (Hare et al., 2015). Based on the stream-bed interface temperature data presented by Rosenberry et al. (2016), Fig. 1 shows how temperaturesensitive fiber optic cables have been used to pinpoint possible groundwater-discharge zones based on an anomalously cold mean temperature and/or reduced thermal variance. A focused evaluation of FO-DTS anomalies with physical seepage meters and vertical temperature profilers confirmed localized, meter-scale seepage zonation along the streambed where discrete colder zones indicated through heat tracing showed approximately 5 times the groundwater-discharge rate of adjacent sandy bed locations only meters away (Rosenberry et al., 2016). The active heating of wrapped FO-DTS cables deployed vertically within an open-valley stream-bed seepage zone indicated the true vertical flow to at least 0.6 m into the bed sediments (Briggs et al., 2016), an expected characteristic of a more regional groundwater discharge (Winter et al., 1998), rather than that of a flow driven by the valley topography local to the river. Hyporheic exchange in the lower Quashnet River system is superimposed on the general upward hydraulic gradient to the stream, therefore being reduced to a thin, shallow hyporheic exchange zone (e.g., < 0.1 m depth) along the thalweg by these competing pressures (Briggs et al., 2014). Vertically compressed hyporheic zones such as these have been simulated for similar stream systems (e.g., Cardenas and Wilson, 2006).

## 3 Methods

**Figure 2.**
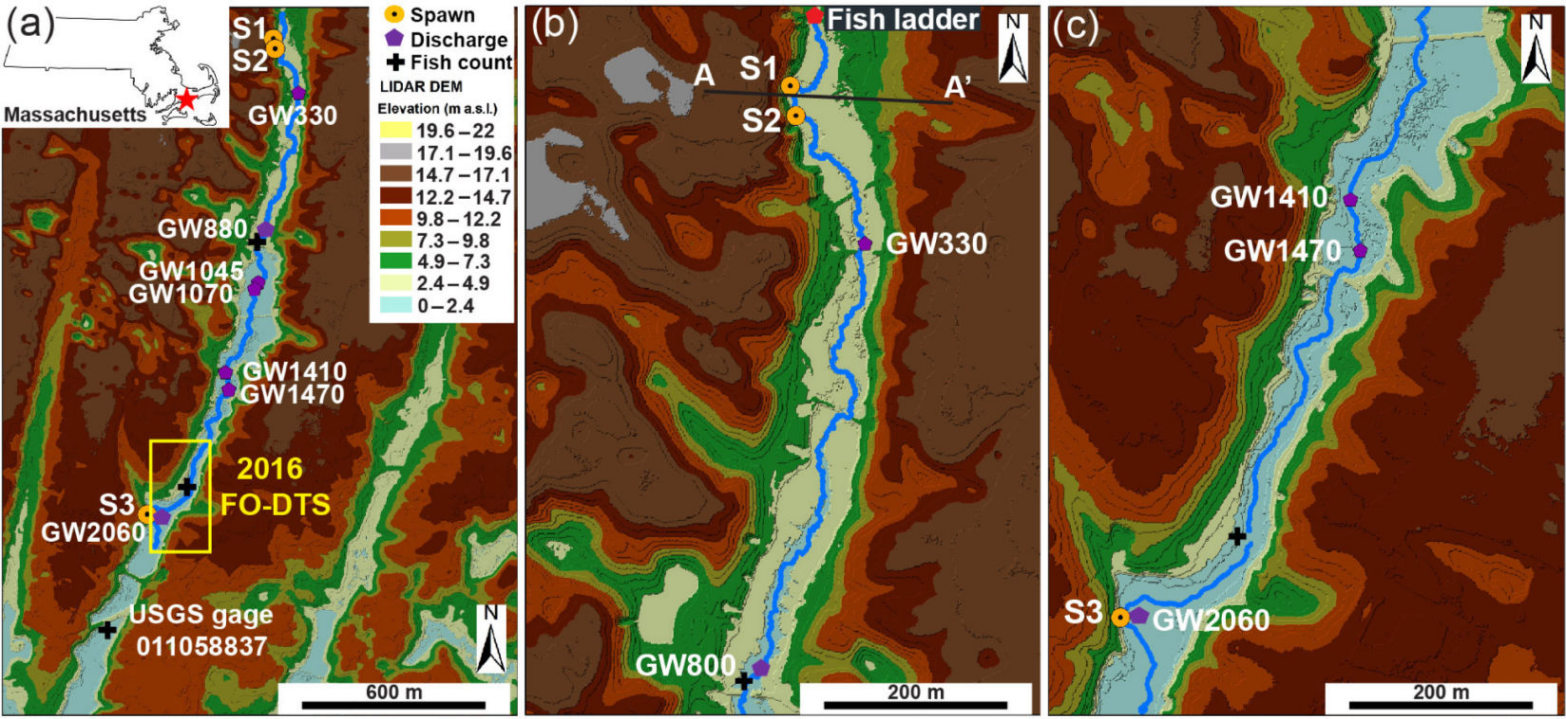
Lidar elevation data show the linear valley terrain of the Quashnet River study reach, as shown in panel (**a**) with Spawn (S1, S2, S3) locations and major open-valley seepage zones identified. The enlarged view of panel (**b**) shows the more narrow upper valley zone where Spawn 1 and 2 are located at the base of a steep cutbank and the topographic transecting point of Fig. 9 (A–A’) is noted. Finally, panel (**c**) displays the lower open-valley reach where Spawn 3 is located along a major cutbank.

A combination of fish tagging and visual spawning observations, heat tracing, geophysical surveys, and focused porewater sampling was used to investigate the interplay between the locations of preferential brook trout spawning and the local hydrogeology. For consistency between varied methods and years of data collection, all sample locations are spatially referenced as downstream channel distances from the fish ladder river crossing at the upper end of the study reach (Fig. 2).

### 3.1 Observations regarding repeat spawning locations

Observations of discrete repeat brook trout spawning locations were made opportunistically as part of an ongoing PIT tagging study of the native reproducing population of the Quashnet River. Large-scale trout movements are continuously monitored in the lower Quashnet River at three stationary fish counting sites (Fig. 2a). However, the spatial resolution of these counting sites, separated by hundreds of meters, is not adequate in studying how brook trout utilize specific decimeter- to meter-scale zones of groundwater discharge. For this finer scale characterization, dropped fish tags have also been located through roving surveys using a handheld portable PIT antenna (Biomark, Inc.), which have been conducted in spring and fall since 2007. The dropout of PIT tags from the fish body is a process that is more likely to happen during spawning behavior in salmonids, so dropped tags were electronically and spatially mapped to reveal discrete zones of repeat spawning. Although these roving surveys do not yield the temporal continuity of the instream counting gates, the clustering of dropped tags can be mapped at the sub-meter scale, presumably directly at trout redds. In addition, spawning brook trout were located visually during annual fall data collection events by Massachusetts Fish and Wildlife Staff, with redd development behavior captured in one seepage feature by an underwater video in 2015 using a GoPro Hero camera (San Mateo, CA). We refer to the three most prominent sites of brook trout spawning within the study reach as Spawn 1 (113 m), Spawn 2 (146 m), and Spawn 3 (2062 m), from upstream to downstream, respectively (Fig. 2).

### 3.2 Spatial mapping of preferential groundwater discharge

To augment existing stream-bed interface thermal surveys for preferential groundwater discharge (e.g., Rosenberry et al., 2016; Fig. 1) and to investigate the bank dependence of the discharge location, ruggedized fiber-optic cables suitable for stream use were deployed in the river along the base of each bank from 1700 to 2160 m on 10 to 12 June 2016 (Fig. 2a). Two separate cables weighted with stainless steel armoring were installed directly along the foot of each bank on top of the stream-bed interface. Single-ended measurements made at the 1.01 m linear spatial sampling scale were integrated over 5 min intervals on each channel by an Oryx FO-DTS control unit (Sensornet Ltd.). During the same period, data were also collected along a high-resolution wrapped fiber-optic array for a dataset described in Kurylyk et al. (2017) but not shown here; this experimental setup resulted in measurements for each channel of four instrument channels, which were recorded at 20 min intervals. The calibration for dynamic instrument drift was performed automatically using approximately 30 m of cable for each channel, submerged in a continuously mixed ice bath and monitored with an independent Oryx T-100 thermistor.

### 3.3 Quantification of vertical groundwater discharge rates

Once preferential discharge locations are located along the stream bed with FO-DTS, actual vertical discharge rates can be assessed using a variety of methodologies (Kalbus et al., 2006). Temporal patterns in the groundwater-discharge flux rate can indicate source flow path hydrodynamics and can be derived from a bed-temperature time series using vertical temperature signal transport characteristics, as reviewed by Rau et al. (2013). Custom “1DTempProfilers” designed specifically for the quantification of groundwater discharge (Briggs et al., 2014) were used to monitor the stream-bed temperature over time along a shallow vertical profile. Profilers were deployed within a subset of the thermal anomalies previously identified with FO-DTS. The profiler deployment locations were chosen to represent a range of preferential groundwater-discharge rates and characteristics based on the on the observed FO-DTS temperature anomalies, e.g., anomalies of the varied mean temperature and buffering effect (Fig. 1) located at 330, 880, 1045, 1070, 1410, 1470, and 2060 m. These groundwater-discharge locations are referred to with the prefix “GW” followed by the meter mark for the remainder of the paper, such that the major stream-bed seep 330 m downstream of the fish ladder is referred to as “GW330”. Data were collected at various locations from 11 June to 13 July 2014, 21 August to 13 September 2015, and 5 June to 9 July 2016. These deployments included the installation of 1DTempProfilers at the near-bank and channel sides of observed repeat spawning zones.

Individual thermal data loggers (iButton Thermochron DS1922L, Maxim Integrated) were waterproofed with silicone caulk and inserted horizontally into short slotted-steel pipes (0.025 m diameter). The shallow thermal profilers were driven vertically into the stream bed so that sensors were positioned at some combination of 0.01, 0.04, 0.07, and 0.11m depths. Data were collected at temporal intervals of 0.5 h in 2014, 0.5 h in 2015, and 1 h in 2016. Rosenberry et al. (2016) found that when a subset of the 2014 stream-bed temperature data presented here were analyzed using the diurnal signal amplitude attenuation models employed by VFLUX2 (Irvine et al., 2015), a near 1 : 1 relation was found in comparison to physical seepage meter measurements of groundwater discharge ranging from 0.5 to 3 m d^−1^. A similar diurnal signal-based stream-bed thermal parameter estimation is used here.

### 3.4 Stream-bed groundwater discharge and spawning zone pore-water characterization

Subsurface water samples were collected for chemical analysis at seven major open-valley seepage locations and three repeat spawn locations. Geochemical data collection occurred in 2014 and 2016 along with the 1DTempProfiler deployments, while stable water isotope data were collected in August 2017. For geochemical sampling, 0.0095 m (nominal) stainless steel drive points were inserted to depths of 0.3,0.6, and/or 0.9 m and Masterflex Norprene tubing was attached to the drive point. A peristaltic pump was used to extract pore-water samples until they were free of obvious turbidity (typically requiring 3 min of pumping), after which the pumping rate was slowed and the groundwater samples were collected by pumping into 60 mL high-density polyethylene (HDPE) syringe barrels. First an unfiltered sample for specific conductivity was pushed from the syringe into a 30 mL HDPE Nalgene sample bottle. Second, a filtered sample for anion analysis was collected after attaching a 0.2 μm pore size (25 mm diameter) Pall polyethersulfone filter to the syringe. Lastly, the pumping rate was slowed again and an overflow cup was attached to the Norprene sample tubing and was held upright until it overflowed, at which point the DO was measured by a field colorimetric test using the manufacturer’s evacuated reagent vials (Chemetrics V-2000). DO concentrations were read twice and the test was repeated using an alternative vial kit if results were near the concentration range limit or out of range. The collected samples were kept cool and out of the light and analyzed for Cl^−^ upon return to the laboratory using standard ion chromatographic techniques.

In addition to the drive point samples, pore-water samples were also collected in June 2016 from shallow depths 0.015, 0.04, 0.08 and 0.15 m below the stream-bed surface at locations GW1045 and Spawn 1, 2, 3 using MINIPOINT samplers (e.g., Harvey and Fuller, 1998). Water was pumped simultaneously from all depths using a multi-head pump that withdrew small-volume samples (15 mL) at low flow rates (1.5 mL min^−1^) to minimize the disturbance of natural subsurface fluxes and chemical gradients. Pumped lines terminated at press-on luer fittings that were pushed onto 0.2 μm pore size (25 mm diameter) Pall polyethersulfone filters. Samples for specific conductivity were collected, whereas filtered samples were collected for anions in pre-labeled 20 mL LDPE plastic scintillation vials with Polyseal™ caps. Sample lines were then attached to overflow cups and dissolved oxygen concentrations were measured as described above.

During a follow-up field effort in August 2017, streambed pore-water samples were collected at the Spawn sites and at GW1045, GW1140 (approximately 70 m downstream of GW1070), and GW1470. Additionally, two large hillslope springs were identified along the edge of the riparian zone, upstream of Spawn 1, using a handheld thermal infrared camera (FLIR T640, FLIR Systems, Inc.). These exposed springs were sampled to identify a localized hillslope groundwater signature that would not be impacted by valley-floor peat deposits. Samples were drawn from push-point piezometers installed 0.2–0.44 m below the sediment interface, with deeper samples collected in the hillslope springs to avoid surface organic material. Pore water was evaluated for specific conductivity (SpC), DO, and stable water isotopes. Isotope samples were analyzed by the U.S. Geological Survey Stable Isotope Laboratory using dual-inlet isotope-ratio mass spectrometry. A substantial fraction of regional Cape Cod shallow groundwater exchanges with the numerous groundwater flow-through lakes as it discharges to the coast (Walter and Masterson, 2002). It is therefore assumed that the regional Cape Cod groundwater isotopic signature is likely to indicate evaporative processes (LeBlanc et al., 2008), offering a contrasting signal from locally recharged hillslope groundwater (no substantial evaporation). The local deuterium excess of contemporary water can indicate groundwater that has been influenced by evaporation in lakes and is therefore in disequilibrium with local meteoric water. Deuterium excess was determined here as *D*_xs_ = *δ*^2^H – 8•*δ*^18^O (Dansgaard, 1964).

As mentioned previously, historic cranberry farming practices extensively modified the Quashnet River valley, including the incision of drainage ditches into the floodplain. Some ditches extend from the valley wall to the main channel, whereas others are shorter or cut at angles. In addition to characterization of pore water, 34 major drainage ditches (observed flowing water) and a stream thalweg profile were spot-checked for specific conductivity on 16 June 2014 using the SmarTroll probe (YSI). At a subset of these ditch locations, filtered grab samples were collected and analyzed in the laboratory for Cl^−^ in a similar manner as the mini and drive point samples described above. In June 2016, the dataset was augmented for five ditch confluence locations upstream of Spawn 1.

### 3.5 Visualizing stream-bed sediment structure

Ground penetrating radars (GPR) have been successfully applied to several surface water and groundwater exchange studies to characterize underlying peat and sandy deposits (e.g., Lowry et al., 2009; Comas et al., 2011) due to strong expected differences in matrix porosity (water content), which can exceed 70% in peat (Rezanezhad et al., 2016). An upstream to downstream GPR profile was collected on 7 July 2016 using a MALA HDR GX160 shielded antenna (MALA GPR, Sweden), towed down the stream center channel by hand with a small inflatable watercraft. The locations of major seep and spawning sites were specifically marked on the digital GPR record during data collection. The GPR data were processed using Reflexw software (Sandmeier, Germany) to convert reflection time to interface depth.

## 4 Results

The hydrogeochemical characterization of observed repeat trout spawning zones and other major stream-bed groundwater-discharge zones are contrasted below.

### 4.1 Observations regarding repeat spawning locations

**Figure 3.**
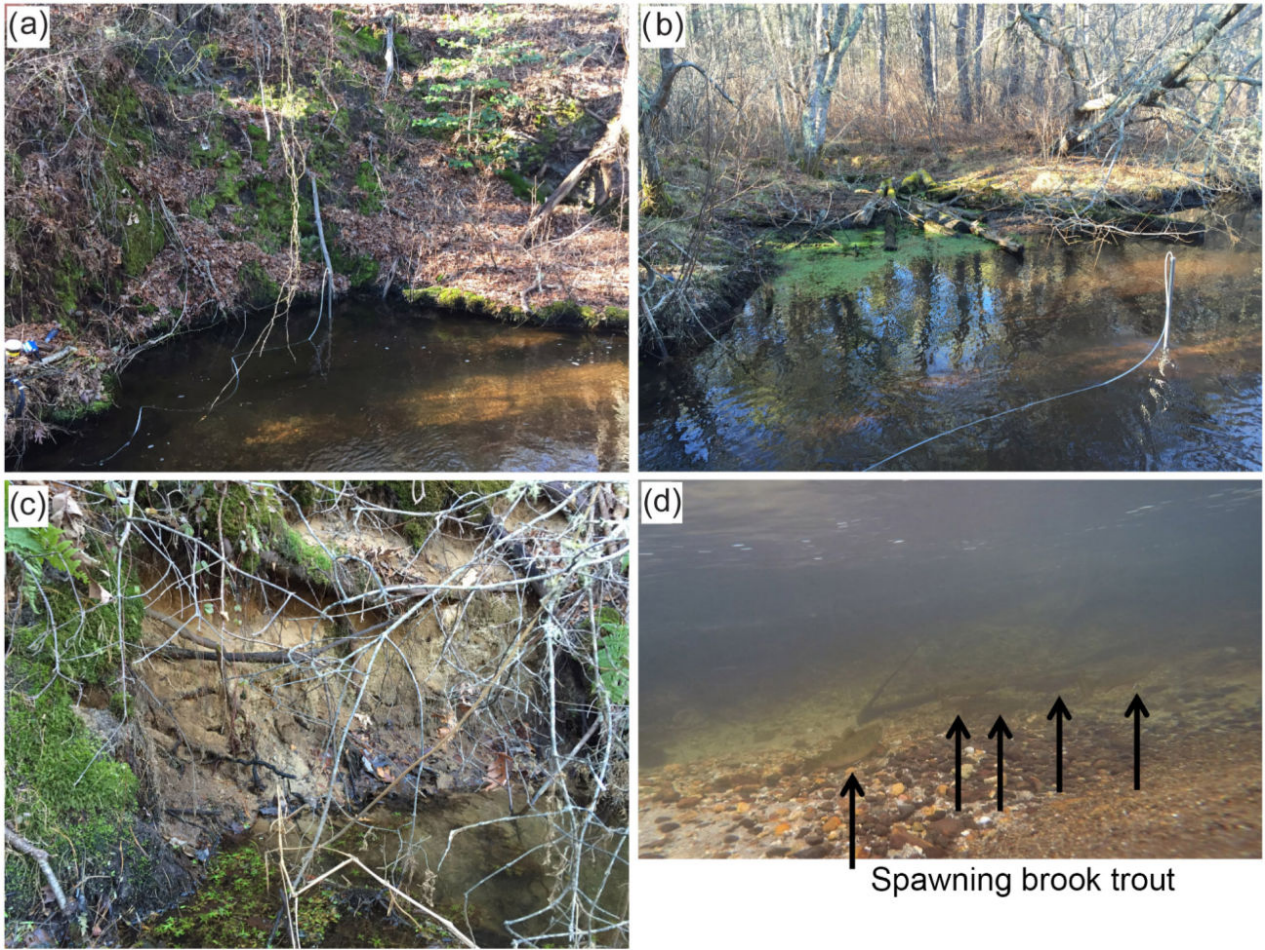
Several representative images of specific spawn zones and groundwater-discharge zones were collected in February 2016. The cutbank alcove at Spawn 1 is shown in (**a**), while the open-valley seepage zone GW1045 is shown in (**b**), and fresh cutbank slumping and visible seepage at Spawn 3 is shown in (**c**). Underwater imagery collected at the Spawn 1 zone in fall 2015 is displayed in (**d**), showing several fish clustered directly at the base of the cutbank where pore-water samples were obtained.

Out of the dozens of preferential groundwater-discharge zones geolocated along the Quashnet River in this and previous work (e.g., Fig. 1), brook trout appear to consistently utilize only three discrete stream-bed locations for repeat spawning activity. These locations coincide with steep cutbanks where the river channel approaches the sand and gravel valley wall (Fig. 2b, c). Specifically, trout were found to occupy small “scalloped” alcove-bank features (Fig. 3a) that may be formed by groundwater sapping of fines and the subsequent slumping of sandy bank materials. In winter 2016, fresh slumping and direct seepage from the newly exposed sand wall was observed at Spawn 3 (Fig. 3c); a larger slump event had filled approximately one-third of the scalloped alcove at Spawn 2 by June 2016. Brook trout were observed clustered along the inner bank area at the Spawn 1 location in fall 2015 (Fig. 3d), and this spawning behavior was captured using an underwater video (Supplement).

Dropout PIT tags have been found repeatedly in each of the three preferential spawn zones. Seven dropout PIT tags were located in the Spawn 3 zone in March 2017, by far the most dropped tags found in any one location since the tracking program began in 2007. The only other obvious scalloped bank features along the 2 km study reach are located at GW1045 (Fig. 3b). Compared to the trout spawning zone alcoves along the valley-wall cutbanks (e.g., Fig. 3a), this open-valley seepage alcove was overgrown with watercress and thick (tens of centimeters), loose deposits of organic material.

### 4.2 Spatial mapping of preferential groundwater discharge

**Figure 4.**
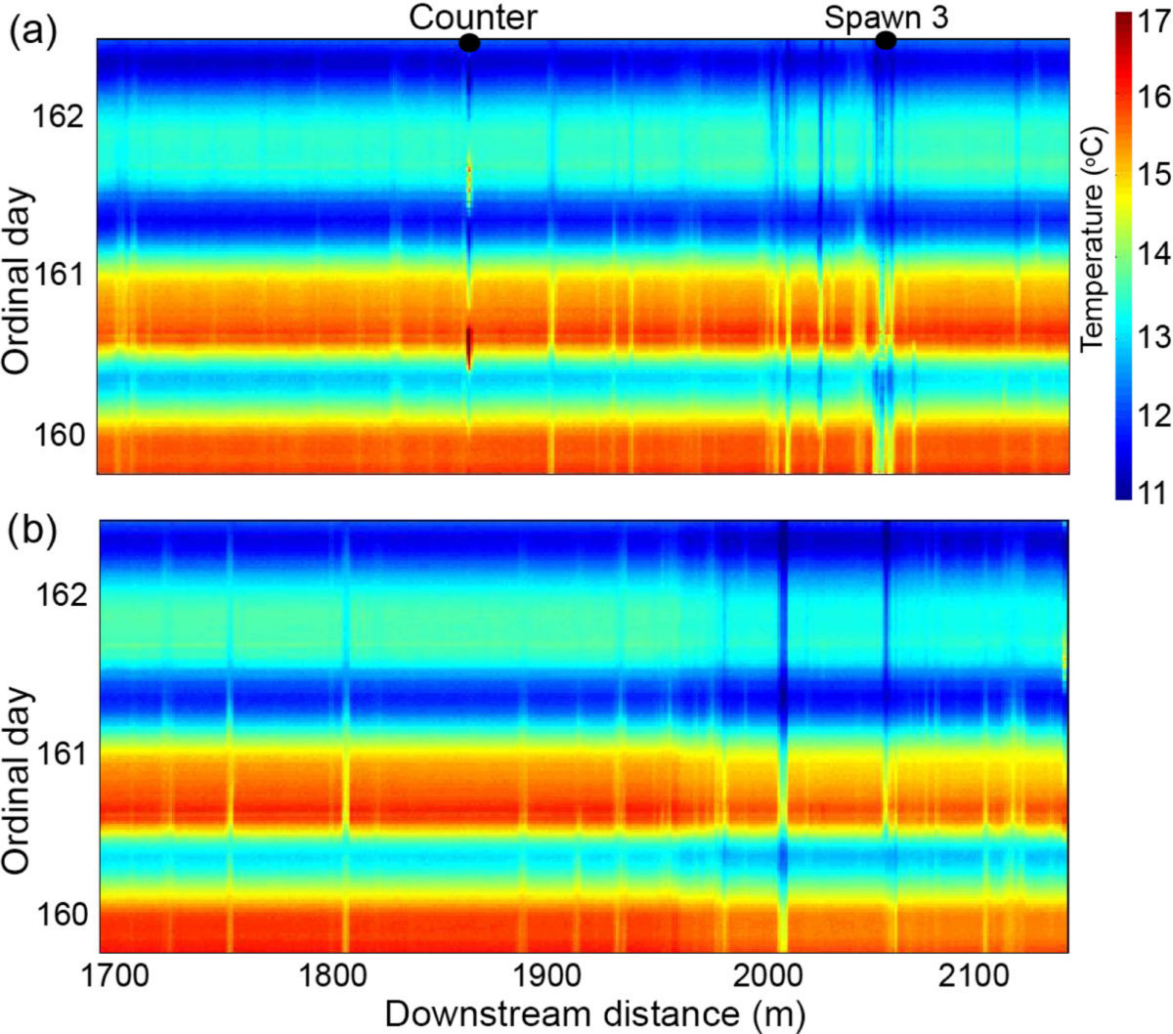
Fiber-optic-distributed temperature data collected from the approximate channel distance of 1700 to 2160m along (**a**) the downstream right bank through the Spawn 3 meander bend area (see Fig. 2a for location), and (**b**) the downstream left bank along the same stream reach. The persistent vertical bands of relatively cool temperatures indicate discrete groundwater discharge. Some larger zones display a thermal signature on both bank cables, while smaller discharges may be specific to one bank.

As shown in Fig. 1, previously collected FO-DTS data were used to guide data collection at a subset of representative preferential stream-bed groundwater discharges. Additionally, paired FO-DTS cables were deployed at the base of both stream banks through a lower reach section in 2016 (Fig. 2c), revealing differing thermal anomaly patterns (Fig. 4; Briggs et al., 2018b). The cable along the downstream-right bank captures a large, 8 m long cooler zone at Spawn 3 (Fig. 4b), and this seepage signature is spatially reduced but visible along the opposing bank (Fig. 4a). Other thermal anomalies observed along one bank show little or no signature along the other. Air temperature dropped noticeably over the final 1.5 days of deployment, and smaller cool anomalies that appeared on warm days were no longer captured by the streambed FO-DTS deployment, though the Spawn 3 signature is still visible along both cables.

### 4.3 Quantification of vertical groundwater-discharge rates

Ambient stream-bed temperature signal data can be used to measure stream-bed thermal conduction parameters (Luce et al., 2013), which is particularly important when applying heat-based methods to quantify upward vertical fluid flux (Rosenberry et al., 2016), compared to downward fluid-flux models that generally show less sensitivity to stream-bed thermal parameters. Diurnal signal-based thermal diffusivity measurements derived from a pair of 1DTempProfilers inserted in sandy channel sediments for a month in 2014 have the same geometric mean value of 0.11 m^2^d^−1^, and this value is used here to model vertical groundwater discharge for all locations and data collection periods (Briggs et al., 2018b). Sub-daily groundwater-discharge fluxes evaluated over similar spring and early summer time periods in 2014 and 2016 show relatively stable patterns at open-valley seepage zones, generally < 1md^−1^ (Fig. 6). At Spawn 1 and 3 seepage is stronger (2 to 3.5 md^−1^) and more variable than at open-valley zones. The Darcy-based horizontal seepage estimate through the Spawn 3 bank, made using the bank piezometer, is 2.3 md^−1^, which is similar to the temperature-based seepage rates at the Spawn 3 interface (Fig. 6), and indicates lateral discharge through the cutbank wall from a more localized groundwater flow path. The Spawn 2 zone shows a reduced and more stable discharge rate during summer 2016, and is likely impacted by a large bank slump into this zone that occurred during the winter of 2016, partially filling the alcove. Seepage patterns collected at Spawn 1 and 2 in late-summer 2015 show greater temporal stability, even though the stream stage at the downstream U.S. Geological Survey gage showed substantial variation. Discharge rates along the inner bank wall of the scalloped bank spawn zones were consistently higher than at bed areas located just a few meters away toward the channel.

### 4.4 Stream-bed groundwater discharge and spawning zone pore-water characterization

Based on previous characterization, the Cape Cod sand and gravel aquifer generally has high DO concentrations (9–11 mg L^−1^), relatively dilute specific conductance (SpC, 62pScm^−1^), and dilute chloride concentrations (Cl^−^, 9.3 mgL^−1^) at depths ranging between 12 and 20 m (Savoie et al., 2012). The groundwater that discharges to the Quashnet River, however, is often strongly variable in all three of these parameters (Harvey et al., 2018). In June 2014, drive point data were primarily collected in open-valley seepage zones identified with FO-DTS (Fig. 1); these locations are suboxic to anoxic at 0.3 and 0.6 m stream-bed depths (Table 1). The highest stream-bed seepage DO is found at GW330 in the tighter upstream valley section (4.6 mg L^−1^ at both depths) and Spawn 3, where DO is 9.0 and 7.6 mg L^−1^ at 0.3 and 0.6 m depths, respectively (Table 1). SpC is also variable, but lowest and similar to the regional signal at GW330 and Spawn 3. Note that SpC and Cl^−^ are used here to indicate aquifer flow path hydrogeochemical properties and not unsuitable spawn habitats based on chemical concentration, as their range is well within general brook trout tolerances.

Drive point data collected at the 0.3 m depth in June 2016, primarily around spawn zones, generally show high DO and relatively low SpC at the interior of Spawn zones 1 and 3 near the cutbank (Table 1). Data collected a few meters toward the main channel from these near-bank spawn locations are reduced in DO with increased SpC. The Spawn 2 data were collected at the toe of the recent large sediment slump that had partially filled the alcove, and DO data are suboxic at 0.3m (3.9mgL^−1^) but more oxygen-rich at 0.9m depth (7.2 mg L^−1^), indicating the potential for shallow stream-bed respiration that removes oxygen from discharging groundwater (assuming vertical flow) in the slumped material. In contrast to the spawn zones, the major open-valley seepage location GW1045 is nearly anoxic at all depths with SpC similar to the 2014 stream water profile grab samples *(n* = 8, 101.4± 1.7 μS cm^−1^). Little difference was observed between near-bank and channel positions at GW1045 (both are sub-oxic) even though a large scalloped seepage bank feature was observed (Fig. 3b).

The drainage-ditch grab samples generally show Cl^−^ concentrations that are lower than the average 2014 channel grab samples (n = 10,19±0.4mg L^−1^), though the two most upstream ditches are similar to stream water, and 2 open-valley ditches are appreciably higher in Cl^−^ (Fig. 7a). Spawn zones 1, 2, and 3 approximate the lowest Cl^−^ concentrations observed in drainage ditches, and Spawn 3 has a similar concentration to the adjacent 2016 stream-bank piezometer in both the 2014 and 2016 data. An analogous pattern is shown in the more widespread SpC data, with many drainage ditches and all spawn zones having concentrations around 60 μS cm^−1^. However, several ditches cluster around the stream water average or higher, particularly in the open-valley area.

The shallow, shallow pore-water samples collected with the MINIPOINT system in discrete intervals show that stream-bed SpC is appreciably lower than stream water, even at the 0.02 m depth, at all near-bank spawn zones (Fig. 8a). Conversely, the shallow channel sediments at Spawn 1 and open-valley seepage at GW1045 approximate the stream water value for SpC. DO is high and stable along the shallow profiles (to 0.14 m) at the interior of Spawn zones 1 and 3 but suboxic at the Spawn 1 channel sample and Spawn 2 zones and essentially anoxic along the bank at GW1045. Center channel pore-water samples at GW1045 show moderate oxygen enrichment at 0.02m (4.6mgL^−1^), which may result from hyporheic mixing, as deeper intervals along the same profile are nearly anoxic.

The underwater video collected here in the fall of 2015 indicates Quashnet River brook trout clustered tightly around an approximate 1 m^2^ bed area in Spawn 1 (Fig. 3d, Supplement), directly at the base of the sandy cutbank. During the June 2016 collection of pore-water data, drive points were installed precisely in this area. A chemical analysis of 0.3 m deep pore water shows a strong gradient from the near-bank Spawn 1 zone to the outer alcove area, with specific conductance rising dramatically (70.6 to 143.9 μS cm^−1^) and DO falling (7.28 to 4.41 mg L^−1^) (Table 1). Spawn 3 shows a similar pattern from the near-bank zone toward the main channel (60.4 to 82.1 μS cm^−1^ SpC; 9.11 to 1.76 mg L^−1^ DO). Spawn 2, although complicated by the large slump during the previous winter, shows an increase in SpC from 70.6 to 139.3 μS cm^−1^ from the inner to outer alcove. Conversely, pore water collected at 0.3, 0.6, and 0.9 m depths in the open-valley seepage alcove at GW1045 (pictured in Fig. 3b) are functionally anoxic with elevated SpC compared to inner spawn zones and have little gradient from the bank to the channel.

Pore-water data collected in August 2017 indicate that all three Spawn sites are similar to emergent hillslope springs, characterized by relatively high DO and low SpC, compared to major open-valley stream-bed seepage zones that are anoxic with higher SpC (Table 2). Additionally, the stable isotopic signatures of the hillslope and Spawn zones are similar, but are contrasted by the lower deuterium excess metric determined for the open-valley seepages. This indicates that groundwater discharging through the stream bed away from the hillslope shows the evaporative signature of groundwater flow-through lakes and can therefore be considered regional discharge, compared to locally recharged hillslope groundwater apparently favored by trout for spawning.

### 4.5 Visualizing stream-bed sediment structure

**Figure 5.**
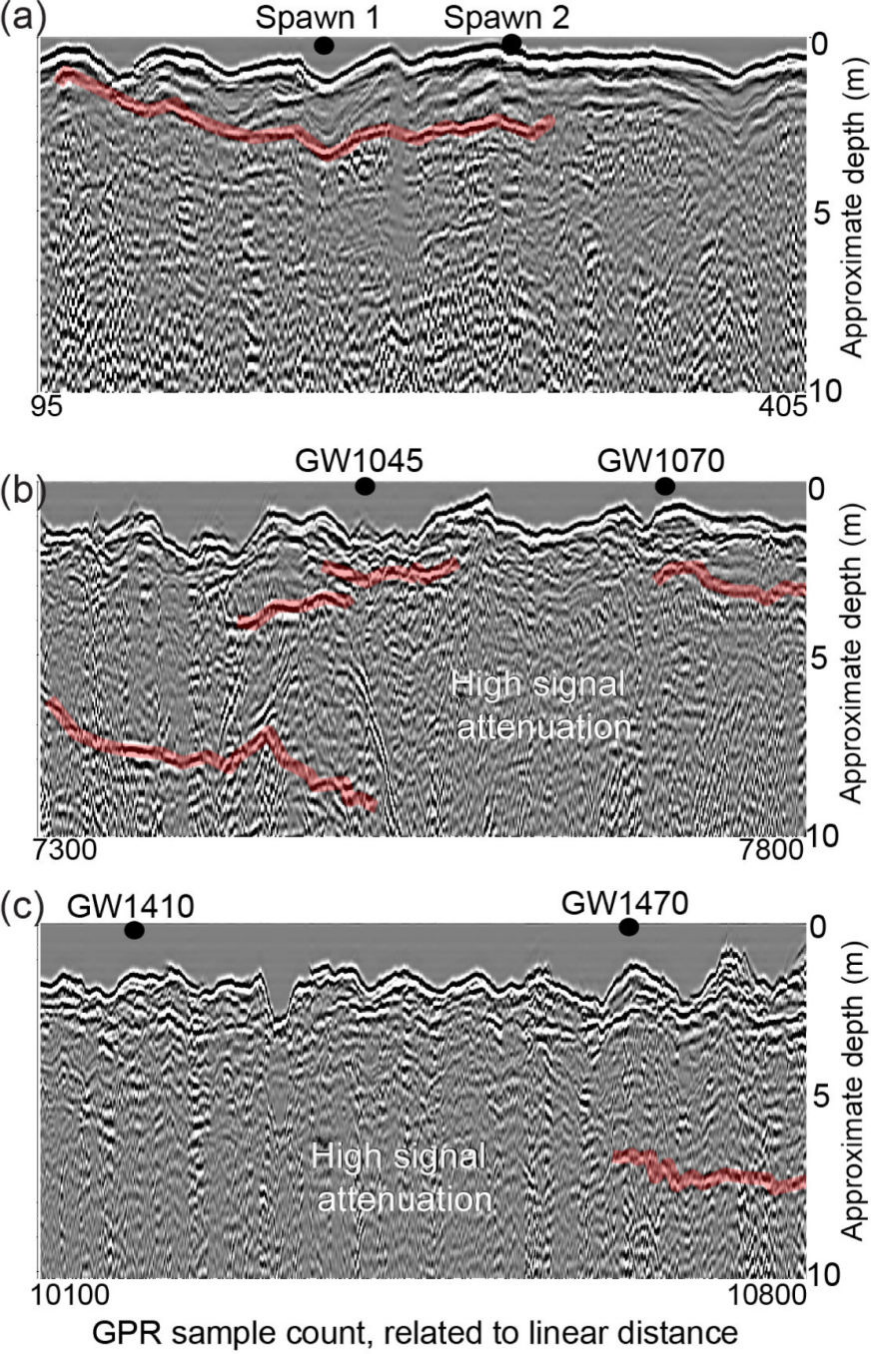
These images show ground-penetrating radar profiles collected down the center of the river channel to indicate peat, sand, and gravel layering in the stream bed. Stronger apparent radar reflectors are highlighted in red and likely indicate sediment layer boundaries (e.g., sand and gravel vs. peat). Spawn- and groundwater-discharge locations were directly marked in the radar data stream during collection and are shown for each sub-reach panel.

Radar data were collected over most of the study reach length depicted in Fig. 2a, and although spatial reference data were not collected for each sample point due to integrated global positioning system failure, Spawn and groundwater-discharge zones of interest were precisely marked in the record (Fig. 5). The GPR data collected along the thalweg adjacent to Spawn 1 and 2 indicate that a contiguous thin layer of material underlies the sandy stream bed that may be peat deposited over deeper sands and gravels (Fig. 5a). The GPR profile through open-valley groundwater-discharge locations GW1045 and GW1070 shows the strongest radar signal reflectors of anywhere along the open-valley section (Fig. 5b). These discontinuous geologic structures are interpreted as layered sand and gravel, interspersed with thicker peat deposits. Otherwise, discontinuous reflections indicative of sediment-type interfaces of variable depths are observed near the downstream open-valley seepage zones and strongly attenuated GPR signals indicate thick lenses of buried peat with high water content (Fig. 5b,c).

## 5 Discussion

Heat tracing reconnaissance technologies, such as FO-DTS and thermal infrared, offer an efficient means to comprehensively characterize preferential groundwater-discharge points at the reach to watershed scale (Briggs and Hare, 2018). Using the groundwater-fed Quashnet River as an example, Rosenberry et al. (2016) showed that cold stream-bed interface anomalies in summer indeed correspond to discrete zones of particularly high groundwater discharge through stream-bed sediments. This spatial characterization of discharge points alone is not sufficient to understand the physical and chemical drivers of a niche habitat, but can efficiently guide additional data collection, as was done here. Compared to more randomly distributed stream-bed field parameter surveys and larger spatial scale evaluations of net groundwater discharge made with differential gaging, the comprehensive spatial mapping of groundwater discharge using heat is a great advance in the context of understanding groundwater-dependent ecosystems. However, in fast flowing streams, FO-DTS cable placement on the stream bed will likely impact which specific groundwater-discharge zones are captured with FO-DTS, as shown here by applying cables along opposite banks through the Spawn 3 area (Fig. 4). The largest seepage zones may have a spatial footprint that encompasses the stream-bed area from bank to bank (e.g., the Spawn 3 cold anomaly), but a subset of more discrete seepage zones are bound to be missed with a single linear cable deployment. We did not capture Spawn zones 1 and 2 in early FO-DTS field efforts (Fig. 1), but fish tracking indicated their importance in regards to trout spawning behavior. Therefore, in studies of niche stream habitats as influenced by preferential groundwater discharge, a combination of heat tracing and biological observation may be needed to both identify major discharge points and discern which points are directly used by the biota of interest (e.g., brook trout).

In a study of the regional Cape Cod aquifer condition, Frimpter and Gay (1979) state that groundwater is typically near DO saturation, except in the case of the downgradient of peat or river bottom sediments, where consumption of DO allows the mobilization of natural iron and manganese. Visible observations along the open-valley section, in addition to stream-bed sediment coring (Briggs et al., 2014), revealed the widespread coating of shallow stream-bed sediment grains with metal oxides, consistent with the conceptual model of organic material influence on near-surface groundwater (Fig. 9). Aquifer recharge passing through upgradient groundwater flow-through kettle lakes (e.g., Stoliker et al., 2016) may also serve to decrease the DO content of the regional flow paths that discharge vertically through the bed of the Quashnet River, although we hypothesize that localized peat deposits may be the primary control on both seepage zone distribution and chemistry.

Out of the dozens of preferential groundwater-discharge zones located along the lower Quashnet with heat tracing, most were suboxic to anoxic (Table 1). Brook trout consistently prefer three areas for fall spawning, all along meander bend cutbanks into the sand and gravel valley wall. Zones of locally enhanced seepage, likely controlled by subtle differences in sediment hydraulic conductivity, can lead to the groundwater sapping of fines, reduction in bank stability, and consequent slumping of bank material into the river; this process was observed in real time at the Spawn 3 meander in February 2016 (Fig. 3c). Slumping effectively forms *seepage-driven* alcoves outside of the main flow and are more suitable for redd placement, along with forming a more favorable coarse sand and gravel substrate (Bowerman et al., 2014; Hausle and Coble, 1976; Raleigh, 1982).

**Figure 6.**
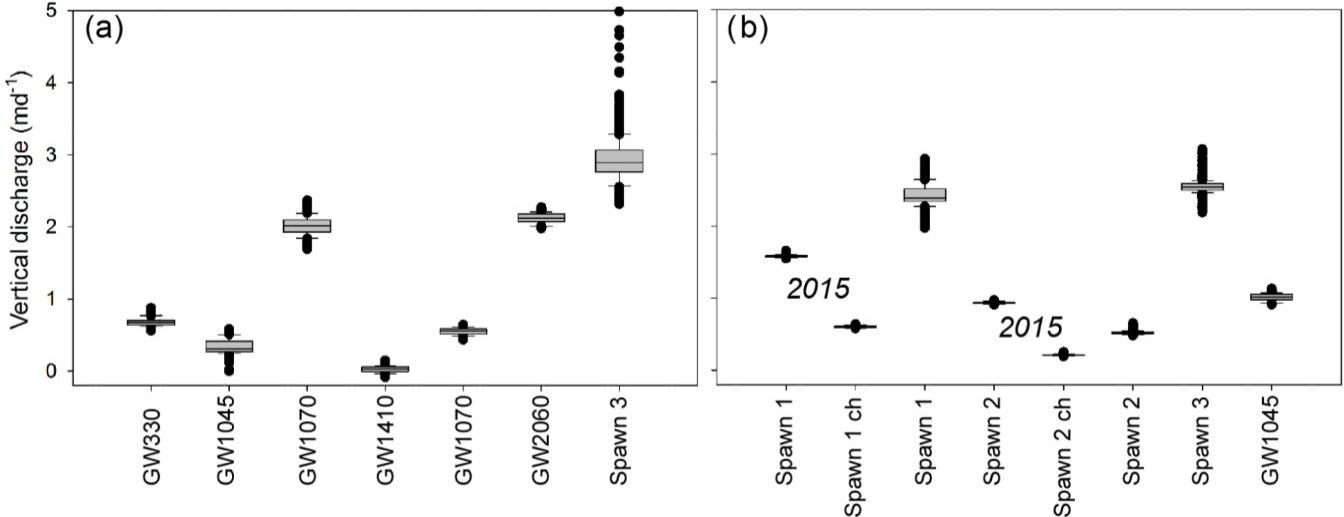
Summarizing box plots of sub-daily vertical groundwater-discharge rates modeled for the open-valley groundwater discharge and Spawn 3 bank locations for the 11 June to 13 July 2014 period are shown in panel (**a**). Additionally, panel (**b**) displays discharge rates collected in Spawn and GW1045 locations directly against the cutbanks and farther out towards the channel (indicated by “ch”) for the 21 August to 13 September 2015 and 5 June to 9 July 2016 periods.

In other systems, trout have been observed to occupy microhabitat around and within groundwater-discharge zones, even being segregated by fish size and desirable temperature range (e.g., Fig. 2.4.1.2 in Torgersen et al., 2012). Here, real-time observation and visual imagery show trout clustering tightly against the bank in Spawn 3 (Fig. 3d, Supplement) where pore water was found to be more oxygen rich and lower in SpC. The month-long time series of vertical groundwater-discharge rates are reduced considerably from the near-bank to the near-channel areas at all spawning zones (Fig. 6), indicating in part a reduction in stream-bed hydraulic conductivity as influenced by peat deposits under the main channel and as observed in GPR data (Fig. 5). The evidence of higher near-bank vertical groundwater flux rates and DO combined with lower SpC indicates limited interaction between the shallow groundwater flow paths and peat against the meander bend cutbanks. As observed in other systems, it appears that even short travel distances through organic deposits toward the center channel at Spawn 1 and 2 may be sufficient in increasing total dissolved solids, depleting DO (e.g., Levy et al., 2016), and rendering upwelling zones undesirable for redd construction. Therefore, near-surface channel sediments may need to be specifically characterized in preferential groundwater-discharge zones, as net chemical reactivity over the last ~ 1 m of transport may dominate net chemical change of the discharging groundwater.

The alcove seepage features utilized by trout in this study are apparently similar to the numerous cold-water alcove patches observed in another stream system by Ebersole et al. (2003). In that study of preferential salmonid habitats, alcoves were often located where streams converged on valley walls and were the most abundant type of discrete cold-water habitat type identified. Conversely, valley-wall alcoves were the least common type of seep morphology observed along the Quashnet River. It is likely that the artificial reduction in channel sinuosity along the Quashnet River by farming practices has reduced the number of natural higher-quality spawning locations.

Other bank and alcove features with strong groundwater discharge found along the open-valley section (Fig. 3b) were highly influenced by organic material deposition and did not apparently support spawning habitats. Our research indicates that in lowland systems with organic-rich floodplain sediments, valley-wall alcoves alone create a favorable brook trout spawning habitat via local mineral soil-dominated groundwater-discharge flow paths, as shown in conceptual Fig. 9. This finding might help inform future ecologically based stream restoration practices in using the natural landscape to predict desirable preferential groundwater-discharge points, as was recently done by Hare et al. (2017) to inform the engineering of a large-scale cranberry bog restoration.

The pore-water SpC, Cl^−^, and DO data alone do not definitively show that seepage at the cutbank spawn sites is derived from more localized groundwater recharge, as opposed to regional groundwater that is unadulterated by buried peat lenses. However, the hydrodynamic data derived from long-term vertical temperature profiling in seepage zones does offer additional insight. In general, groundwater-discharge rates are more variable at cutbank spawn zones than in the open-valley stream-bed zones (Fig. 6), and this variability may be tied to shorter-term changes in local river stage and/or water table depth, impacting the local hydraulic gradient. The relatively stable patterns of open-valley groundwater discharge may be controlled by the regional gradient, where the flow path length term dominates the Darcy relation and is therefore relatively insensitive to local changes in river stage and water table fluctuations. Furthermore, the stable water isotope data display evaporative signatures at the open-valley stream-bed discharge sites, indicating regional groundwater that has passed through one or more upgradient flow-through lakes (Table 2). In contrast, the Spawn sites all show isotope signals that fall along the local meteoric waterline and therefore likely represent recharge to the hillslopes more local to the river. These localized groundwater flow systems would be expected to be less influenced by regional groundwater contamination, which is widespread in the regional Cape Cod aquifer (Walter and Masterson, 2002).

**Figure 7.**
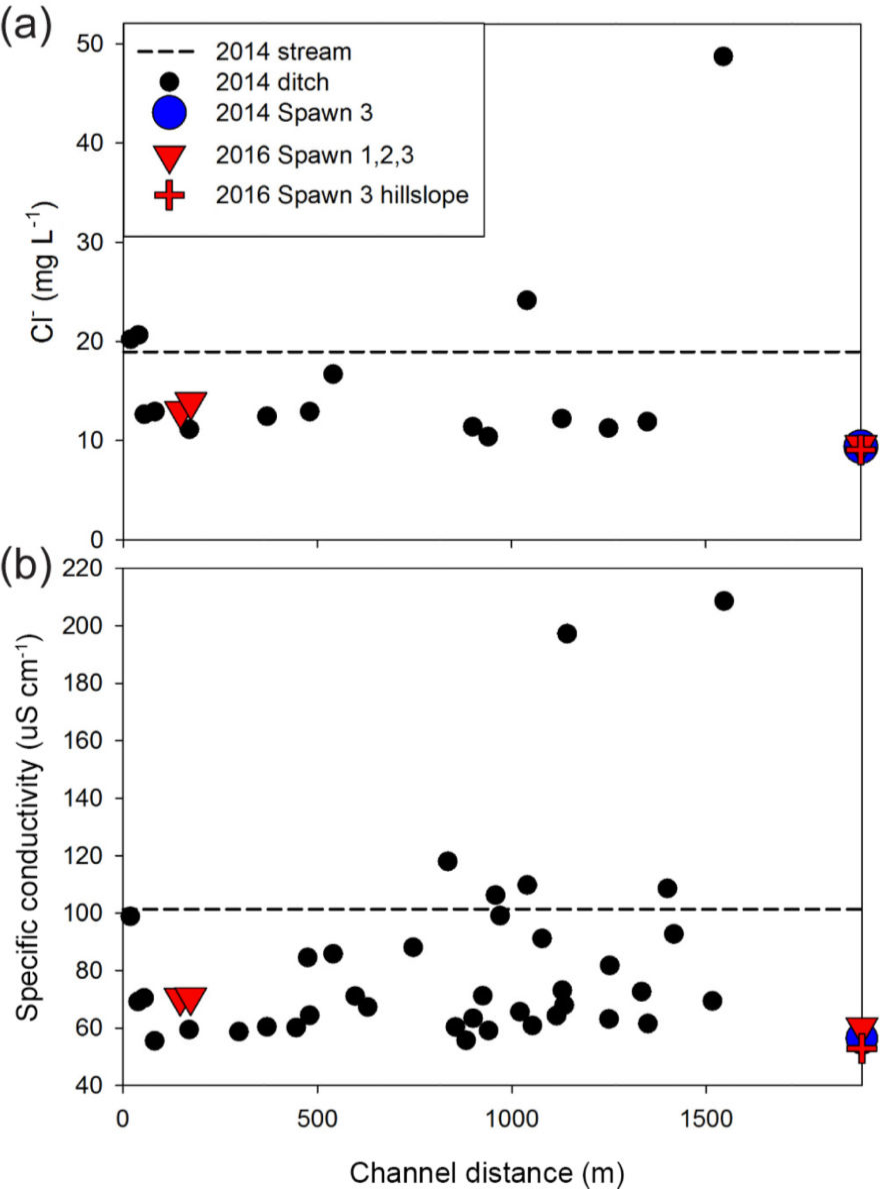
Drainage ditch chemistry throughout the lower Quashnet, showing **(a)** Cl^−^ and **(b)** specific conductance that was collected in June 2014, just above the confluence with the main channel. Data are plotted as the distance from the upper flood control structure in the narrow valley reach and are compared to groundwater-seepage data collected in preferential spawning locations and a hillslope piezometer.

**Figure 8.**
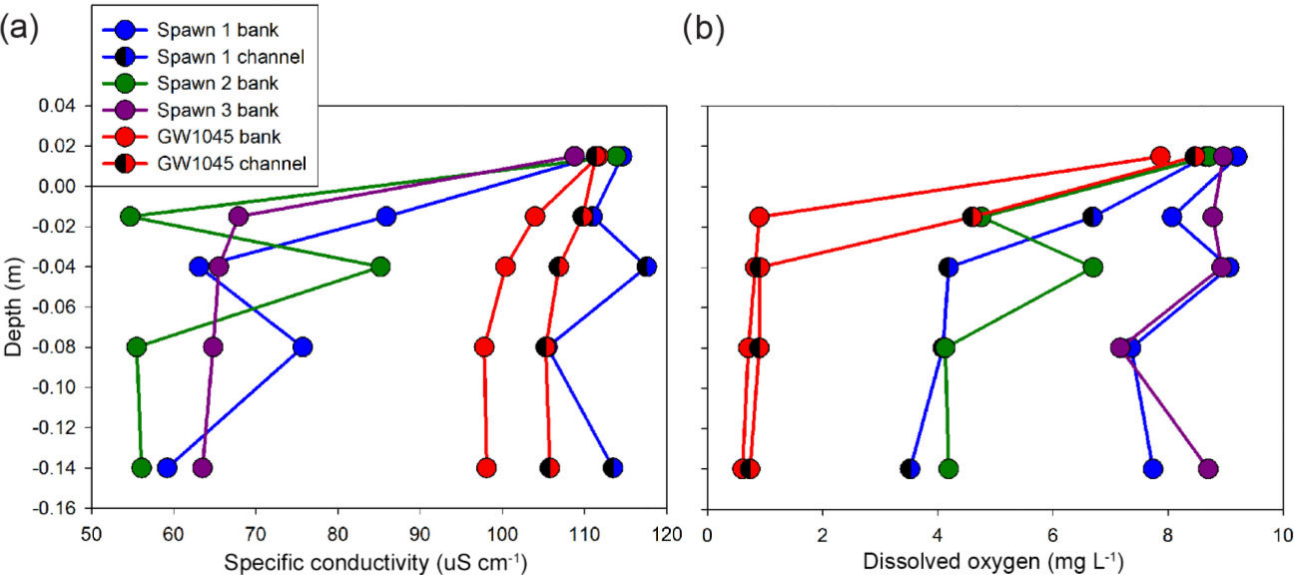
Minipoint pore-water chemistry data showing high spatial resolution profiles of (**a**) specific conductance and (**b**) dissolved oxygen, collected in June 2016 at the major seepage alcoves. Triangle symbols indicate data collected farther toward the thalweg from the respective alcove bank, and all profiles include a local stream water sample taken just above the stream-bed interface.

Groundwater drainage-ditch data collected along the river corridor indicate that low SpC/Cl^−^ conditions exist for the majority of ditches throughout the lower Quashnet River riparian areas (Fig. 7). The hillslope piezometer in sand and gravel at the down valley wall has a similar chemical signature along with high DO. This similarity further indicates that low-SpC groundwater discharges even to the lower portion of the river corridor but is chemically modified by travel through near-stream organics. The relic drainage ditches allow the discharging groundwater to effectively short-circuit the valley floor peat deposits and remain high in DO, similar to the natural valley-wall springs and cutbank alcoves. Future restoration strategies that seek to actively enhance groundwater discharge (e.g., Kurylyk et al., 2015a) may consider capitalizing on this short circuit behavior, possibly by auguring through buried stream-bed peat or through the movement of the stream channel toward the valley wall to create more desirable brook trout aquatic habitat.

## 6 Conclusions

**Figure 9.**
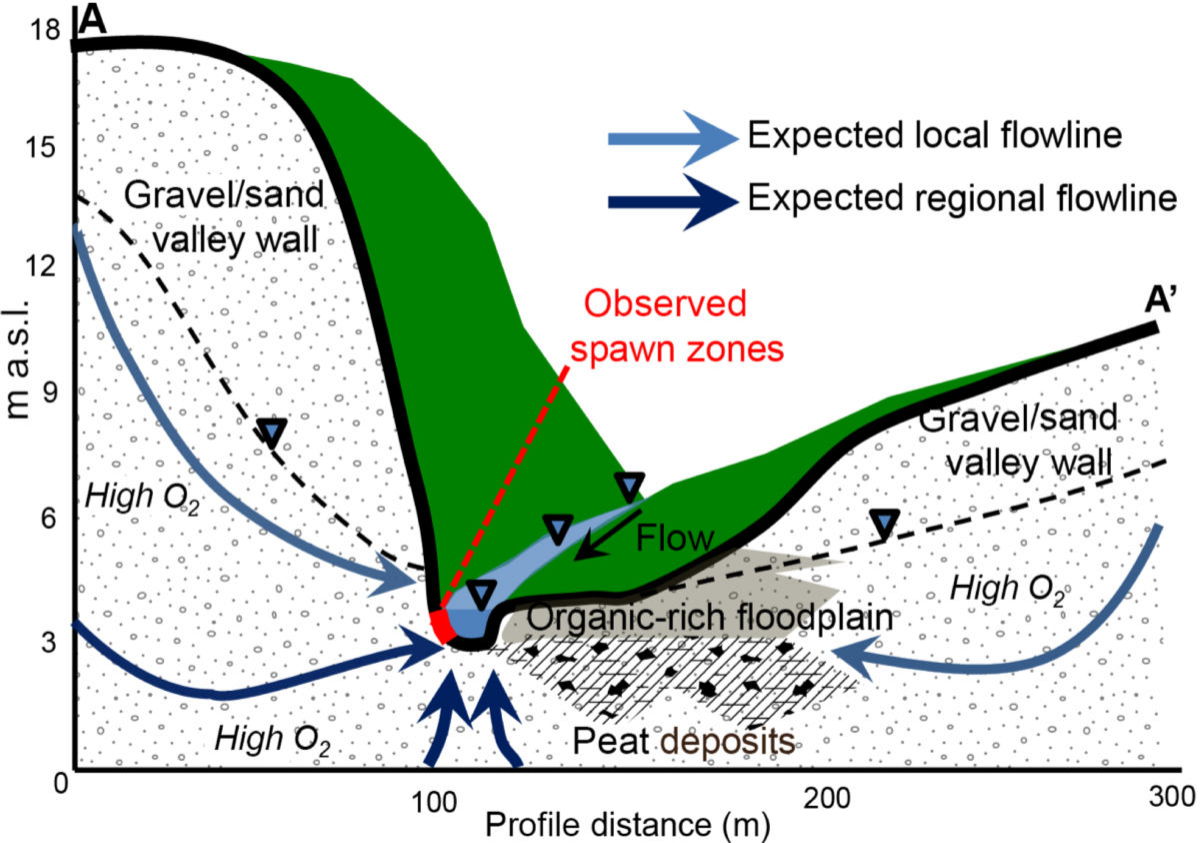
This conceptual model shows how valley-wall cutbank discharge zones are likely sourced by locally recharged hillslope groundwater that avoids substantial interaction with valley-floor organic material. The discharging groundwater remains oxygen-rich, therefore supporting trout spawning activity along discrete stream-bed sections at the meter scale. The topographic profile shown here (A–A’) is derived from airborne lidar data and is oriented perpendicular to the stream at the Spawn 1 zone, as geolocated in Fig. 2b.

The three repeatedly utilized discrete spawning zone locations that have been identified for over a decade of observation have coupled strongly discharging groundwater with high DO concentration. A conceptual diagram of the hydrogeochemical setting of spawn zones vs. other non-favorable stream-bed locations of groundwater discharge is shown in Fig. 9. Spawn zones are located exclusively in side alcoves of the channel created by bank slumps along meanders, where the river cuts into steep hillslopes along the glacial sands and gravel valley wall. In the alcoves at the base of the cutbanks, hillslope groundwater with high DO concentrations is discharged through the stream bed without appreciable loss of oxygen. Just a few meters away toward the main channel, however, groundwater consistently discharges at lower rates, reduces in DO, and increases in SpC. The lowest oxygen concentrations in groundwater are associated with water emerging from the stream bed adjacent to the wide riparian areas that flank the Quashnet in the open-valley section of the study reach, even though groundwater-discharge rates were also relatively high. In the open valley, where the stream is not near the valley walls, proximity to the stream bank does not seem to control seepage chemistry, and GPR data indicated thick zones of discontinuous stream-bed peat. In this and other groundwater-dominated streams that are expected to serve as climate refugia for future native trout populations, hyporheic exchange will be limited by a strong upward hydraulic gradient. Therefore, preferential spawning habitat in such lowland valley systems may be primarily supported by discrete zones of oxic groundwater upwelling at the meter to sub-meter scale, as has been indicated by previous work (e.g., Curry et al., 1995).

In systems where all groundwater discharge is universally anoxic, preferential salmonid spawning zonation may be controlled by points of downwelling hyporheic water where shallow sediments remain high in DO (Buffington and Tonina, 2009; Cardenas et al., 2016). However, these hyporheic areas will deliver cold surface water to shallow sediments during winter, which may impair the overwintering of brook trout eggs (French et al., 2017). Here and in many other coastal systems, groundwater temperature is expected to range from approximately 10–12 °C, which is an ideal range for brook trout egg development (Raleigh, 1982). Points of oxic groundwater upwelling devoid of near-stream buried organics, combined with a recirculating side alcove and favorable sand and gravel sediments, may provide an ideal and unique and preferential spawning habitat for native trout.

Stream surface or stream-bed interface heat tracing of groundwater discharge offers an efficient means to locate discrete seepage zones but offers only limited insight into source groundwater flow path hydraulics and geochemistry. A combined toolkit that also includes spatially informed (using heat tracing) geochemical and isotope sampling and geophysical imaging can be used to trace groundwater flow paths back into the source aquifer, and develop a robust hydrogeochemical characterization. Additionally, as digital elevation models become more refined and combined with infrared data derived from unmanned aerial systems, the remote identification of relatively small features such as the seepage alcoves described here should be possible. A comprehensive and process-based characterization of a niche stream habitat can be used to guide a stream ecological restoration design that directly incorporates the local preferential groundwater-discharge template.

## Supplementary Material

Supp Info

## Figures and Tables

**Table 1.** This table lists 2014 and 2016 drive point pore-water chemistry data collected in major stream-bed groundwater-discharge zones located with fiber-optic heat tracing and in zones of observed repeat trout spawning directly along the bank and farther toward the stream center channel. The italicized values indicate sample depths that differ from others in the same column.

Open valley groundwater discharges	0.3 m depth		0.6 m depth	
	DO mg L^−1^	SpC μS cm^−1^	DO mg L^−1^	SpC μS cm^−1^
GW330	4.6	53.8	4.6	61.3
GW880	1.4	97.7	3.4	65.1
GW1045	0.1	78.8	0.0	82.5
GW1045 (bank)	0.16	105.5	0.39	104.0
GW1045 (channel)	0.31	99.1	0.18	96.4
GW1070	0.2	100.0	0.2	89.8
GW1410	0.0	77.7	0.0	79.0
GW1470	0.1	69.1	0.0	64.3
GW2060	1.4	75.0	0.5	79.4
mean	0.9	84.1	1.0	80.2

Spawning locations (channel)	0.3 m depth		0.9 m depth	

Spawn 1 channel	4.41	143.9	5.68	143.2
Spawn 2 channel	5.25	139.3	n/a	n/a
Spawn 3 channel	1.76	82.1	2.68	79.9
mean	3.8	121.8	4.2	111.6

Spawning locations (bank)	0.3 m depth		0.9 m depth	

Spawn 1 bank	7.28	70.6	9.76	55.9
Spawn 2 bank	3.89	70.8	7.17	57.6
Spawn 3 bank (2016)	9.11	60.4	4.91	71.9
Spawn 3 bank (2014)	9.0	56.4	7.6 *(0.6m)*	60.9 *(0.6m)*
mean	7.3	64.6	7.4	61.6

n/a: not applicable.

**Table 2.** This table lists 2017 drive point pore-water chemistry and stable water isotope data collected in a subset of major stream-bed groundwater-seepage zones, zones of observed repeat trout spawning, and from springs located above the waterline along the same hillslope as the meander cutbanks of Spawn 1 and Spawn 2.

Location	Sample depth (m)	SpC (μS cm^−1^)	DO (mg L^−1^)	δ^2^H (*‰*)	δ^18^O (*‰*)	*D*_xs_ δ^2^H – 8•δ^18^O
Hillslope 1	40	74.82	5.004	−51.38	−8.2	14.22
Hillslope 2	44	60.59	9.318	−51.81	−8.73	18.03
Spawn 1	20	72.45	6.853	−48.9	−7.9	14.3
Spawn 2	20	51.75	5.419	−48.2	−7.95	15.4
Spawn 3	20	42.62	9.054	−44.32	−7.33	14.32
GW1045	20	109.8	0.043	−34.03	−4.93	5.41
GW1140	20	103.4	0.043	−32.56	−4.8	5.84
GW1470	20	97.68	0.04	−33.05	−4.72	4.71
