# Supplementary material for: Hydrogeochemical controls on brook trout spawning habitats in a coastal stream
Source: Hydrol Earth Syst Sci. Author manuscript; Available in PMC 2019 May 14. (PMC6516499; doi:10.5194/hess-22-6383-2018)
Supplement: Supp Info [file NIHMS1022817-supplement-Supp_Info.zip › hess-22-6383-2018-supplement-title-page.pdf]

Supplement of Hydrol. Earth Syst. Sci., 22, 6383–6398, 2018
https://doi.org/10.5194/hess-22-6383-2018-supplement
© Author(s) 2018. This work is distributed under
the Creative Commons Attribution 4.0 License.

Hydrology and Earth System Sciences
Open Access

*Supplement of*

# Hydrogeochemical controls on brook trout spawning habitats in a coastal stream

**M. A. Briggs et al.**

*Correspondence to:* Martin A. Briggs (mbriggs@usgs.gov)

- hess-22-6383-2018-supplement-title-page.pdf
- underwater_trout_spawning.MP4

The copyright of individual parts of the supplement might differ from the CC BY 4.0 License.
